# Homocysteine Induces Collagen I Expression by Downregulating Histone Methyltransferase G9a

**DOI:** 10.1371/journal.pone.0130421

**Published:** 2015-07-20

**Authors:** Wenjing Lei, Yanjun Long, Shuang Li, Ze Liu, Fengxin Zhu, Fan Fan Hou, Jing Nie

**Affiliations:** State Key Laboratory of Organ Failure Research, National Clinical Research Center of Kidney Disease, Division of Nephrology, Nanfang Hospital, Southern Medical University, Guangzhou, P.R. China; Temple University School of Medicine, UNITED STATES

## Abstract

Hyperhomocysteinemia (HHcy) leads to several clinical manifestations including hepatic fibrosis. Excess deposition of extracellular matrix (ECM) components including collagen is the eponymous lesion of liver fibrosis. In this study, we demonstrated that elevated concentration of Hcy induced the expression of collagen type I in cultured human liver cells as well as in liver tissue of HHcy mice. Meanwhile, Hcy inhibited the expression of histone methyltransferase G9a. Mechanistically, silencing endogenous G9a by siRNA enhanced the promoter activity of *COL1A1* in LO2 cells. Conversely, overexpressing G9a inhibited the promoter activity of *COL1A1*. CHIP assay demonstrated that G9a binds to the neuron-restrictive silencer element (NRSE) on the promoter of *COL1A1*. Hcy treatment decreased the binding of G9a on NRSE, which in turn decreased the level of H3K9me2 on the promoter of *COL1A1*, led to upregulation of *COL1A1*. Taken together, these results provide a novel mechanism on explaining how HHcy promotes ECM production.

## Introduction

Homocysteine (Hcy) is a sulfur containing amino acid that is formed as a primary intermediate in the methionine cycle [1]. Methionine from dietary sources is converted to S-adenosyl methionine (SAM) by the enzyme methionine adenosyltransferase (MAT). SAM donates its methyl group to methyl acceptors including phospholipids, DNA, RNA, and protein, and then converted to S-adenosyl homocysteine (SAH), which is then hydrolyzed to Hcy and adenosine in hepatic tissue. Hcy can either be remethylated to methionine or converted to cystathionine by cystathionine- β-synthase (CBS) [2]. Elevated plasma Hcy concentrations, a condition known as hyperhomocysteinemia (HHcy) [3], is the consequence of enzymatic deficiencies and/or nutritional defects that interfere with the proper metabolism of methionine and/or Hcy [4]. The most common genetic cause associated with severe HHcy is homozygous CBS deficiency which is characterized by very high levels of plasma total Hcy (tHcy>200μM). The clinical manifestations of HHcy are very diverse, including mental retardation, cardiovascular problems, skeletal abnormalities, and hepatic compromise, with fatty accumulation and cirrhosis.

Hepatic fibrosis is a wound-healing response characterized by accumulation of extracellular matrix (ECM). In normal liver, ECM is a highly dynamic substratum with a precisely regulated balance between synthesis and degradation. During chronic liver injury, hepatic fibrosis develops as ECM production exceeds ECM degradation. Moreover, the composition of matrix is changed from collagens IV and VI to collagens I, III and fibronectin. There has been tremendous progress in revealing the regulatory mechanisms that control ECM-related gene expression during fibrosis, and research has focused primarily on transcriptional control pathways. TGF-β1 has been regarded as the most important growth factor implicated in collagen synthesis in hepatic fibrosis. TGF-β1 signals via its cognate receptors to Smad proteins to enhance collagen I gene transcription [5,6]. In addition, AP-2, NF-1 and c/EBP has been shown to regulate collagen gene expression [7].

Elevated Hcy levels have long been known to be linked to liver disease [8]. Fatty liver is a common finding in nutritionally induced HHcy due to methionine overload or folate deficiency. Patients with CBS deficiency also have hepatic steatosis which is accompanied by perisinusoidal or central venous fibrosis and fibrosis of hepatic arterioles [9]. It has been shown that CBS-deficient mice develop inflammation, fibrosis, and hepatic steatosis [10,11]. The upregulation of collagen I has been detected in the liver of CBS deficient mice and HHcy rats induced by chronic Hcy administration [12]. However, the molecular mechanism responsible for HHcy-induced collagen expression is unclear. Increased Hcy elevates the level of SAH which inhibits transferring methyl group from SAM to acceptors, leads to hypomethylation [13]. It has been proposed that Hcy-induced DNA hypomethylation is a biochemical mechanism by which Hcy induces vascular injury [14,15]. However, whether epigenetic modifications such as DNA methylation and histone modifications are involved in the pathogenesis of Hcy-induced hepatic fibrosis is largely unknown.

Neuron-Restrictive Silencing Factor/Repressor Element Silencing Transcription Factor (NRSF or REST) is a transcriptional regulator that regulates a network of genes by binding a 21–23 bp neuron-restrictive silencing element (NRSE) [16], which in turn recruits a silencing complex of chromatin remodeling proteins including SIN3A, histone deacetylases 1 and 2 [17], CoREST [18], methyl-CpG-binding protein 2, and histone methyltransferase G9a [19,20]. NRSF was originally perceived as a silencer of neuron-specific genes [17]. Later studies revealed that there are over 800 NRSE-containing genes in human genome, underscoring the importance of NRSF in controlling large programs of transcriptional regulation [19].

In the present study, we found a NRSE in the promoter of both human *COL1A1* and mouse *Col1α1*, and G9a binds to the NRSE. Upon Hcy stimulation, the expression of G9a is decreased, results in reduced binding of G9a to the NRSE, which in turn decreased the level of H3K9me2 on the promoter of human *COL1A1* and mouse *Col1α1*, led to upregulation of collagen I both *in vitro* and *in vivo*. This finding provides a novel epigenetic mechanism on explaining how elevated Hcy promotes liver fibrosis.

## Results

### Hcy induces collagen I expression in cultured normal human liver cells

**Fig 1:**
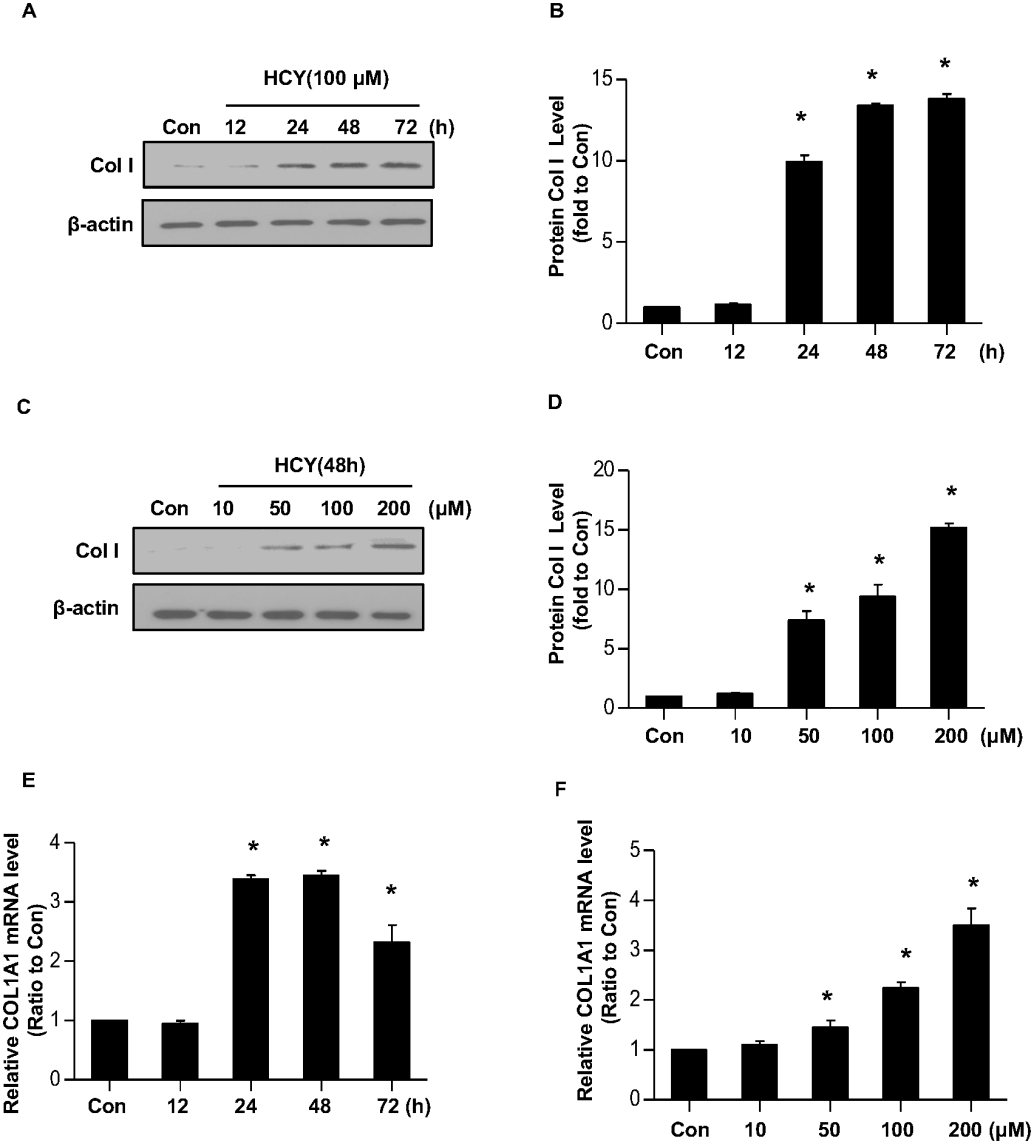
Hcy induces Col I expression in LO2 cells. LO2 cells were incubated with 100μM of Hcy for indicated time period, or indicated concentration of Hcy for 48 h. The protein level of Col I was determined by Western blotting (A-D). β-actin was used to verify equivalent loading. Graphic representation of relative protein level of Col I normalized to β-actin (B, D). The mRNA level of *COL1A1* gene was determined by real time PCR (E-F). Data are mean±SD of three independent experiments. *p<0.05 versus control cells.

Previous study has shown that HHcy causes liver fibrosis [10,21]. We thus examined whether Hcy affects the production of Col I, a major component of ECM. To this end, normal human liver cells (LO2) were incubated with Hcy (100μM) for indicated time period and then cell lysates were harvested for Western blotting. Fig 1A and 1B showed that the protein level of Col I was significantly increased in a time-dependent manner. We further treated LO2 cells with incremental concentrations of Hcy from 10 to 200μM for 48 h. As shown in Fig 1C and 1D, the level of Col I increased progressively with increased concentrations of Hcy. At 200μM of Hcy, the protein level of Col I was ~15 fold higher than that of untreated cells.

Hcy could regulate Col I production at mRNA or post-translation level. To determine this, we examined the transcript of human *COL1A1* in LO2 cells after Hcy treatment by real time PCR. As shown in Fig 1E and 1F, the mRNA level of *COL1A1* was gradually increased in a time- and dose-dependent manner after Hcy treatment, indicating that Hcy upregulates *COL1A1* gene transcription in cultured human liver cells.

### Hcy induces *Col1α1* expression *in vivo*

**Fig 2:**
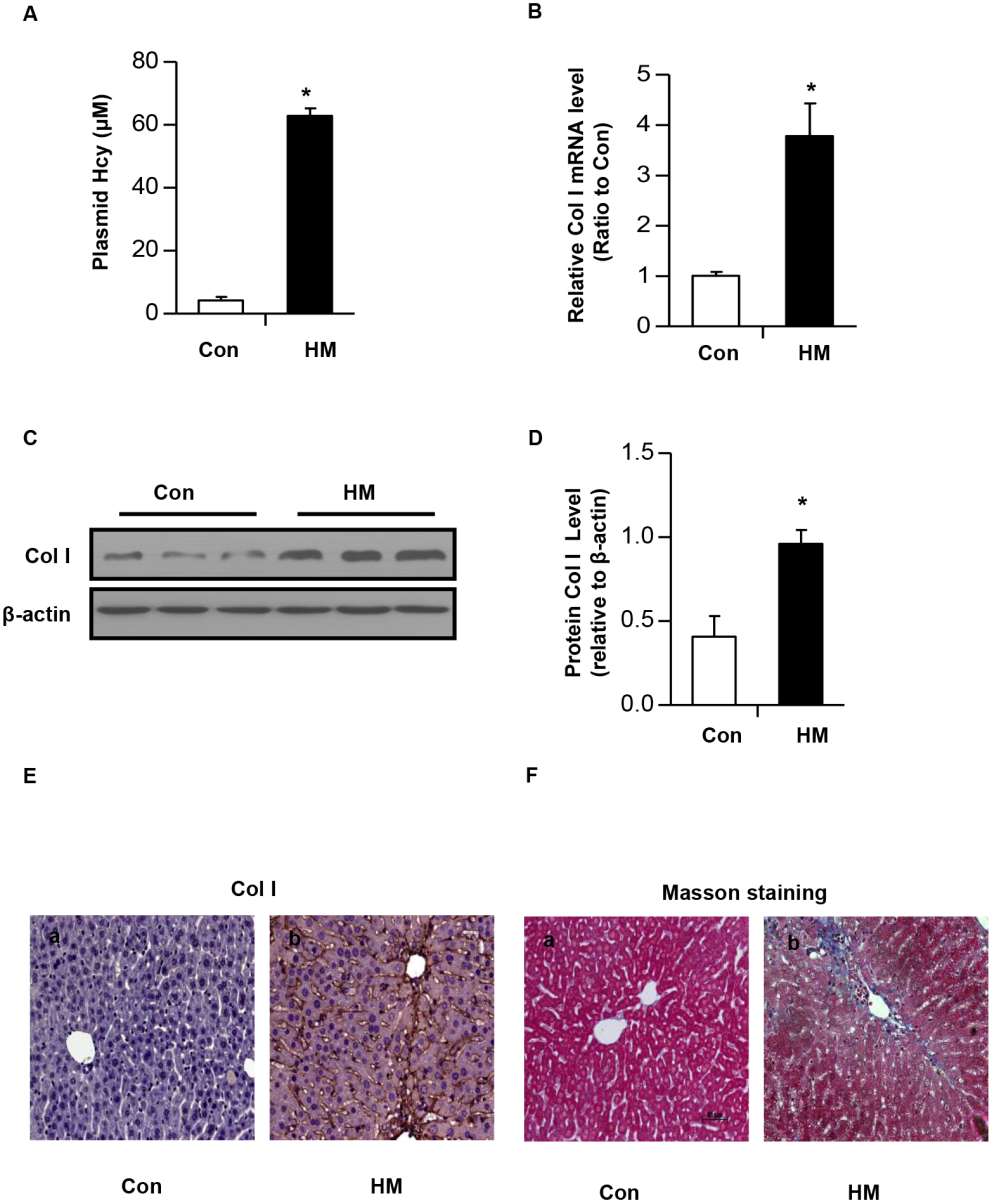
Increased Col I expression in liver of HHcy mice. (A) C57BL/6 mice were fed with regular rodent chow or a high methionine diet (HM) for two weeks, and then liver tissues were collected. (A) The plasma concentration of Hcy in mice. (B) The mRNA level of *Col1α1* gene was determined by real time PCR. (C) The protein level of Col I was determined by western blotting. β-actin was used to verify equivalent loading. (D) Graphic representation of relative protein level of Col I normalized to β-actin. Data are expressed as mean±SD, n = 6. *p<0.05 versus mice fed with regular rodent chow. (E) Immunohistochemical staining for Col I in the liver of HHcy mice. (F) Representative micrographs show that HHcy caused hepatic fibrosis. Liver sections from either control mice or HHcy mice were subjected to Masson’s trichrome staining. Scale bar, 50μm.

To further confirm the effect of Hcy on Col I production *in vivo*, we generated HHcy mice by feeding male C57BL/6 mice with a high methionine diet (HM) containing sufficient basal levels of B vitamins [22,23] for two weeks. The plasma level of Hcy was increased to 67μM±8.7μM, whereas the plasma level of Hcy is 5.5μM±0.7μM in control mice fed with regular rodent chow diet (Fig 2A). Liver tissues of HHcy mice and control mice were collected. Real time PCR showed an upregulation of *Col1α1* gene (Fig 2B). Consistently, Western blotting and immunohistochemistry staining demonstrated that the protein level of Col I were significantly increased in the liver of HHcy mice (Fig 2C–2E). To confirm that HHcy causes hepatic fibrosis, Masson’s trichrome staining was performed. As shown in Fig 2F, compared with that of control mice, a dramatic increase in ECM accumulation was observed in the liver tissue of HHcy mice, which is consistent with previous studies [10].

### G9a mediates Hcy-induced *COL1A1* gene expression

**Fig 3:**
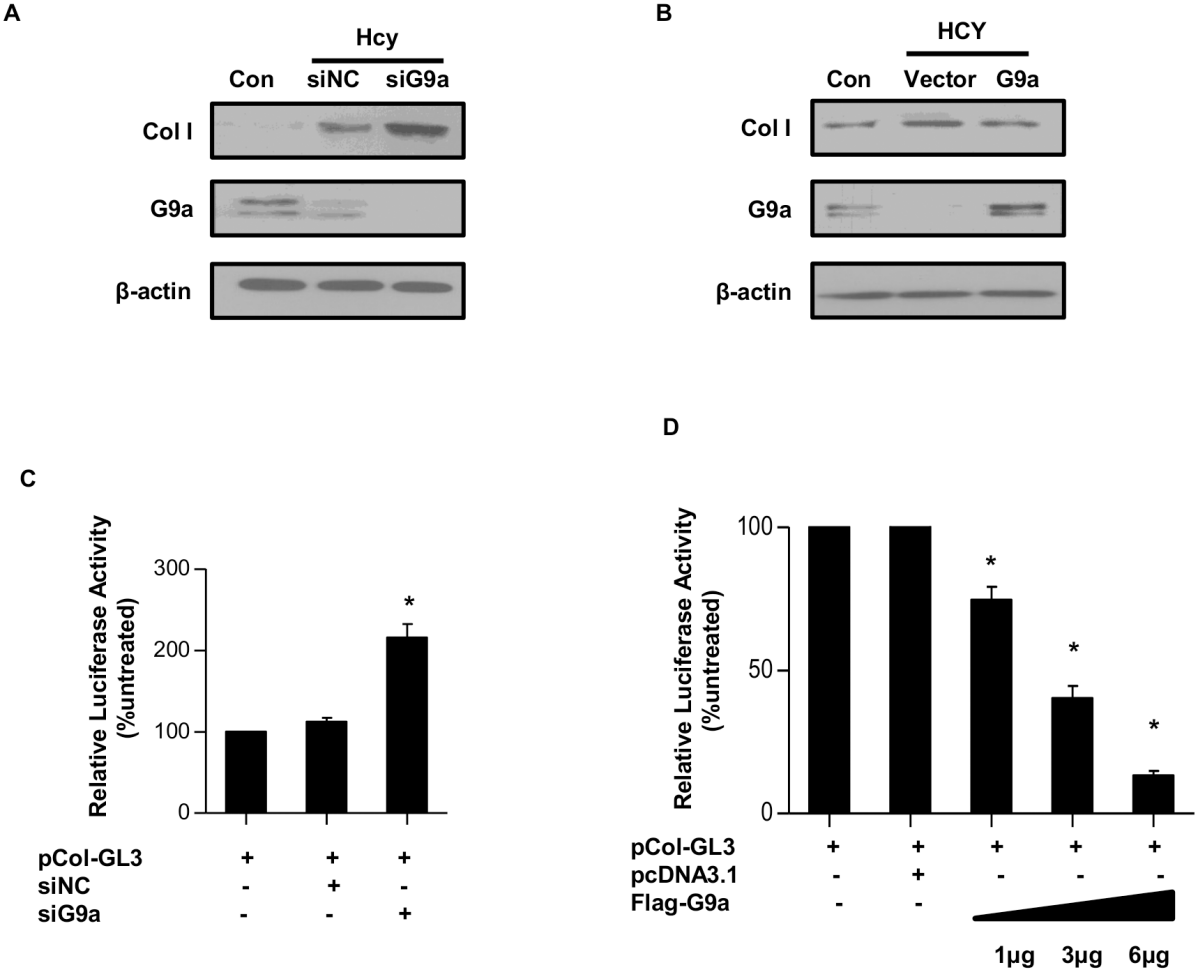
Hcy-induced Col I expression is mediated by G9a. (A) LO2 cells were transfected with scramble siRNA (siNC) or siRNA targeting G9a (siG9a) and then treated with 100μM of Hcy for 48 h. Whole cell lysates were harvested, the protein levels of G9a and Col I were determined by Western blotting. β-actin was used to verify equivalent loading. (B) LO2 cells were transfected with Flag-tagged G9a or empty vector, and then treated with 100μM of Hcy for 48 h. The protein level of G9a and Col I was determined by Western blotting. β-actin was used to verify equivalent loading. (C) LO2 cells were transfected with either siNC or siG9a together with pCol I-luc plasmid and pRL. Relative luciferase activity was presented. *p <0.05 versus siNC transfected cells. (D) LO2 cells were transfected with empty vector or indicated amounts of Flag-tagged G9a together with pCol I-luc plasmid and pRL. Relative luciferase activity was presented. *p <0.05 versus empty vector transfected cells. Data are mean±SD of three independent experiments.

Since we have demonstrated that Hcy modulates *COL1A1* gene expression, we determined to further explore the underlying mechanism. We assessed the promoter regions of both human *COL1A1* and mouse *Col1α1* gene using the online prediction service of NCBI and found a neuron restrictive silencing element (NRSE), which has been shown to recruit G9a to silence gene expression [19,20]. To evaluate the role of G9a in Hcy-induced *COL1A1* gene expression, endogenous G9a was knocked down by siRNA (siG9a) in LO2 cells. LO2 cells were transfected with siG9a or control siRNA (siNC) and then treated with Hcy (100μM) for 48 h. Western blotting revealed that silencing endongenous G9a significantly increased the protein level of Col I when compared with control siRNA trasnfected cells (Fig 3A).

Alternatively, Flag-tagged G9a or empty vector was transfected into LO2 cells followed by Hcy treatment for 48 h. As shown in Fig 3B, Hcy significantly increased the protein level of Col I in empty vector transfected cells. Whereas, the protein level of Col I was significantly lower in G9a overexpressed cells than that of control cells (Fig 3B).

To examine whether G9a directly affect the promoter activity of *COLA1* gene, we synthesized a reporter plasmid bearing a 1-kb fragment of the 5’-flanking region of human *COL1A1* gene (pCol-GL3) and co-transfected it with siRNA targeting G9a (siG9a) or scramble siRNA (NC-siRNA). Compared with NC-siRNA, siG9a significantly enhanced the luciferase activity by 40% (Fig 3C). Next, pCol-GL3 was co-transfected with increasing amounts of Flag-tagged G9a into LO2 cells. As shown in Fig 3D, the promoter activity was gradually decreased as the amount of G9a plasmid was increased. A significant decrease was detected when 1μg of Flag-tagged G9a was transfected. These results support a role of G9a in modulating *COL1A1* gene expression.

### Hcy reduces the binding of G9a to the promoter of *COL1A1*

**Fig 4:**
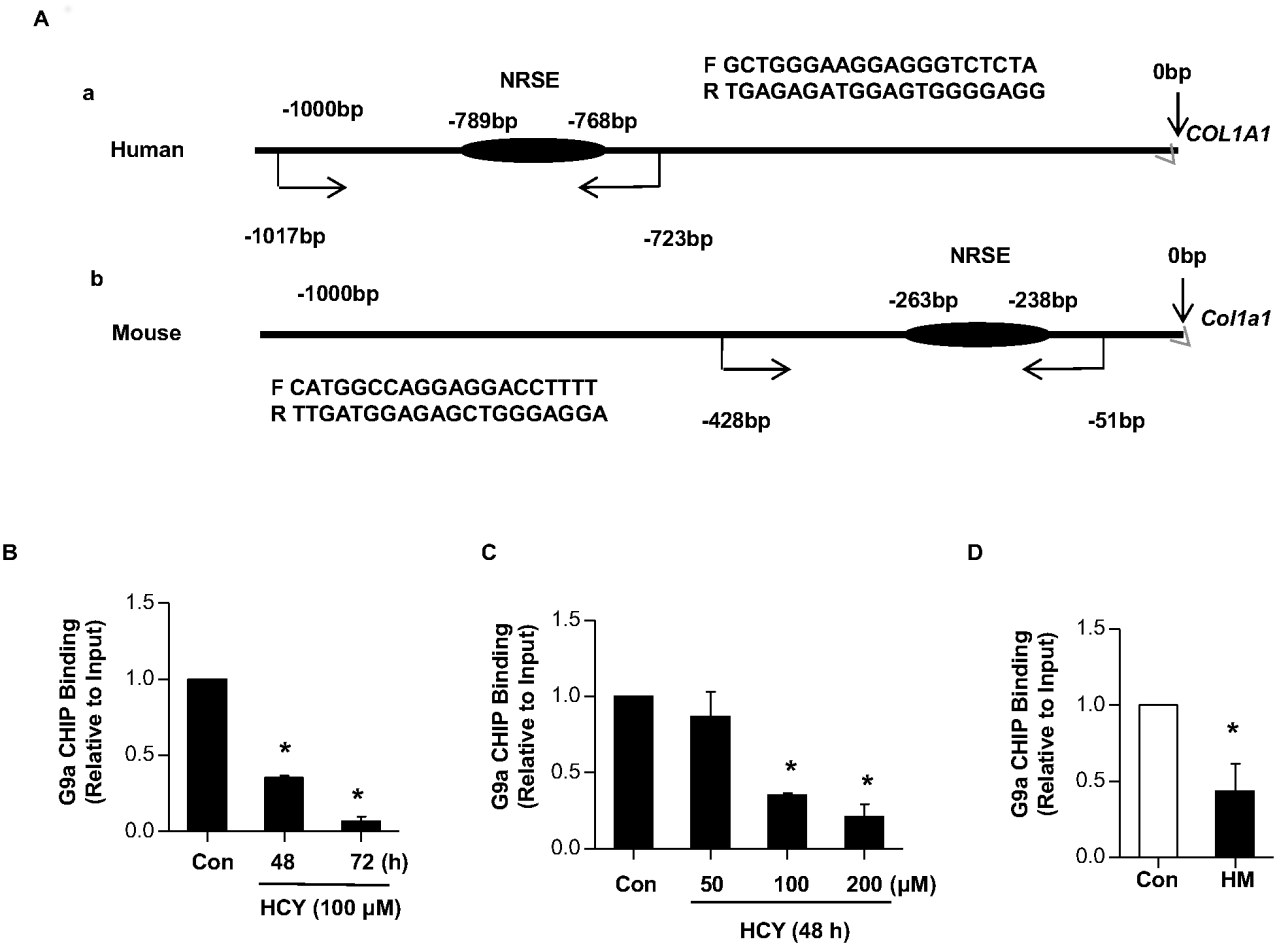
Hcy decreased the binding of G9a to the *COL1A1* promoter. (A) Schematic representation of the promoters of *COL1A1* and *Col1α1* gene. NRSE region and primers used for ChIP assay were marked. (B-C) LO2 cells were treated with 100μM of Hcy for indicated time period (B), or indicated concentration of Hcy for 48 h (C), and then harvested for ChIP assay by using anti-G9a antibody. The changes in G9a on the *COL1A1* promoter were examined by q-ChIP PCR. Data are means±SD of three independent experiments. *p<0.05 versus untreated cells. (D) Liver tissues of wild type mice and HHcy mice were collected for ChIP assay by using anti-G9a antibody. The changes in G9a on the *Col1α1* promoter were examined by q-ChIP PCR. Data are expressed as mean±SD, n = 6. *p<0.05 versus mice fed with regular rodent chow.

Since G9a overexpression inhibits the activity of *COL1A1* promoter and there is a NRSE within the promoter region of *COL1A1*, we hypothesized that Hcy might reduce the binding of G9a to the NRSE on the promoter of *COL1A1*. To evaluate this hypothesis, chromatin immunoprecipitation (ChIP) assay was performed to determine the chromatin occupancy of G9a in the promoter of *COL1A1* using anti-G9a antibody in LO2 cells and mice. ChIP-enriched DNA samples were analyzed by quantitative PCR (qPCR) using primers spanning human NRSE DNA sequences (Fig 4A). To evaluate the effect of Hcy on the binding activity of G9a, LO2 cells were treated with 100μM of Hcy for indicated time period. ChIP assays revealed that binding of G9a to the promoter of *COL1A1* was decreased in a time-dependent manner (Fig 4B). Furthermore, we treated LO2 cells with indicated amount of Hcy for 48 h. Fig 4C showed that the binding of G9a to the promoter of *COL1A1* was gradually decreased in a dose-dependent manner. Similarly, the binding of G9a to the promoter of *Col1α1* was also significantly decreased in the liver of HHcy mice (Fig 4D). Collectively, these data indicate that a decrease of G9a binding to the promoter of *COL1A1* might play a role in Hcy-induced *COL1A1* expression.

### Hcy reduces the level of H3K9me2 on the promoter of *COL1A1*

**Fig 5:**
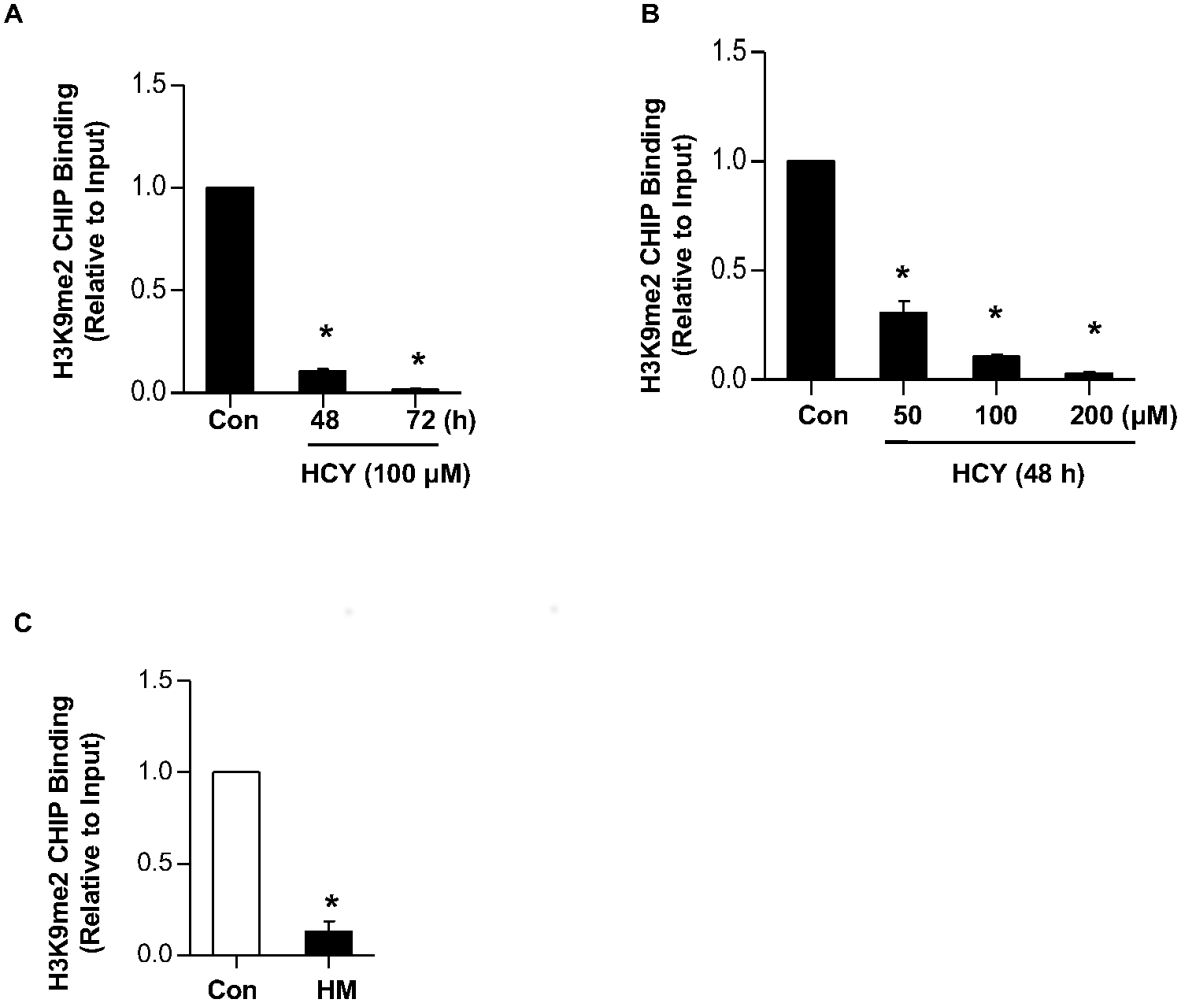
Hcy decreased the level of H3K9me2 on the *COL1A1* promoter. (A) (A-B) LO2 cells were treated with 100μM of Hcy for indicated time period (A), or indicated concentration of Hcy for 48 h (B), and then harvested for ChIP assay by using anti-H3K9me2 antibody. The changes of H3K9me2 on the *COL1A1* promoter were examined by q-ChIP PCR. Data are means±SD of three independent experiments. *p<0.05 versus untreated cells. (C) Liver tissues of wild type mice and HHcy mice were collected for ChIP assay by using anti-H3K9me2 antibody. The changes in H3K9me2 on the *Col1α1* promoter were examined by q-ChIP PCR. Data are expressed as mean±SD, n = 6. *p<0.05 versus mice fed with regular rodent chow.

Since G9a is the primary enzyme for dimethylation of histone H3 lysine 9 (H3K9me2), we next examined whether histone H3 lysine methylation levels were also altered by Hcy. ChIP assay using H3K9me2-specific antibodies revealed that Hcy decreased the level of H3K9me2 on *COL1A1* promoter in a time- and dose-dependent manner (Fig 5A and 5B). Similarly, the level of H3K9me2 on *Col1α1* promoter was also dramatically decreased in the liver of HHcy mice (Fig 5C). These results suggest that upregulation of Col I might be caused, at least partially, by a loss of repressive epigenetic histone modification on its promoter.

### Hcy downregulates G9a expression both *in vitro* and *in vivo*

**Fig 6:**
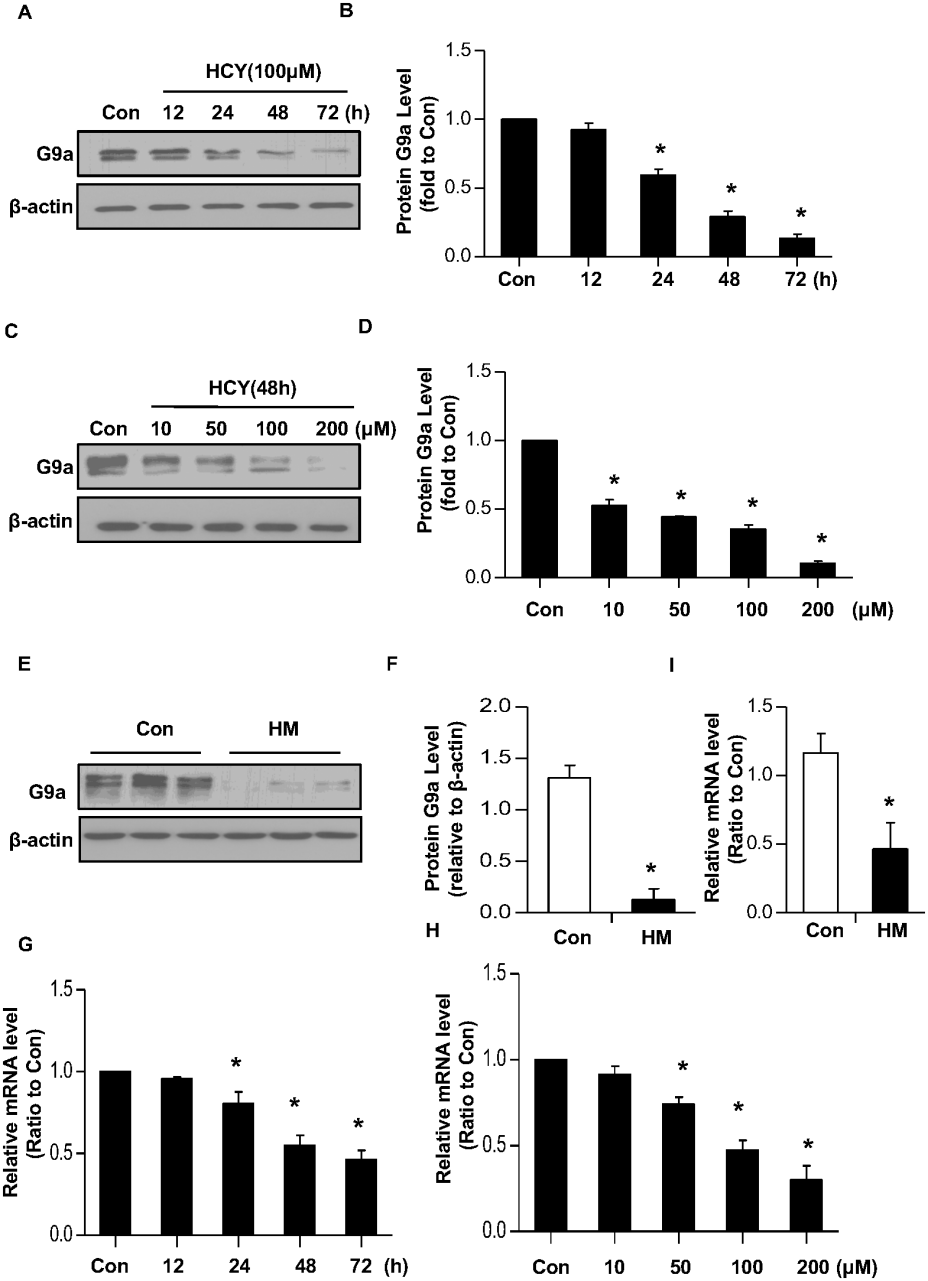
Homocysteine decreases G9a expression both *in vitro* and *in vivo*. LO2 cells were incubated with 100μM of Hcy for indicated time period (A-B), or indicated concentration of Hcy for 48 h (C-D). The protein level of G9a was determined by Western blotting. β-actin was used to verify equivalent loading. Graphic representation of relative protein level of G9a normalized to β-actin (B, D). Data are mean±SD of three independent experiments. *p<0.05 versus control cells. (E) Liver tissues of HHcy mice were collected from HHcy mice or control mice fed with regular rodent chow. The protein level of G9a in liver was determined by Western blotting. β-actin was used to verify equivalent loading. (F) Graphic representation of relative protein level of G9a normalized to β-actin. (G-H) LO2 cells were incubated with 100μM of Hcy for indicated time period (G), or indicated concentration of Hcy for 48 h (H). The mRNA level of G9a was determined by real time PCR. (I) Liver tissues were collected from HHcy mice or control mice fed with regular rodent chow. The mRNA level of G9a in the liver was determined by real time PCR. Data are expressed as mean±SD, n = 6. *p<0.05 versus control mice fed with regular rodent chow.

The decreased binding of G9a to the promoter of both human *COL1A1* and mice *Col1α1* might be caused by a reduced protein level of G9a. To test this possibility, LO2 cells were treated with various concentrations of Hcy for indicated time period. Western blotting revealed that Hcy gradually decreased the protein level of G9a in a time- and dose-manner (Fig 6A–6D). Furthermore, the protein level of G9a in liver tissue of HHcy was also significantly lower than that of control mice (Fig 6E and 6F).

To examine whether Hcy inhibits G9a expression at the transcription level, LO2 cells were treated with various concentrations of Hcy with indicated time period and then mRNA was extracted for real time PCR. As shown in Fig 6G and 6H, the mRNA level of G9a was gradually decreased in a time- and dose-dependent manner after Hcy treatment. Moreover, the mRNA level of G9a in the liver tissue of HHcy mice was also significantly lower than that of control mice (Fig 6I), indicating that Hcy suppresses the transcription of G9a gene.

## Discussion

Gene regulation by extracellular stimuli involves not only transcription factors binding to their cognate DNA binding sites but also epigenetic changes in chromatin [24]. The transcriptional mechanisms on the overproduction of ECM during the process of organ fibrosis have been extensively studied. Collagen I is a downstream target gene of TGF-β/Smads pathway and its expression is induced by TGF-β in fibrotic organs [25]. Previous study showed that CBS-deficient mice (tHcy 205μM) develop liver fibrosis concomitant with an enhanced expression of Col I and TGF-β [8,9], suggesting Hcy-induced TGF-β expression is a mechanism for *Col1α1* gene induction. In the present study, we provided evidence that the transcription of Col I gene is regulated by histone methyltransferase G9a. ChIP assay demonstrated that G9a binds to the promoter of human *COL1A1* and mice *Col1α1*, catalyzes local H3K9me2 to maintain Col I expression at low level under normal condition. Whereas, Hcy downregulates G9a expression, which in turn decreased the level of H3K9me2 at the promoter of *COL1A1*, leading to *COL1A1* gene induction. This finding suggests that changes of histone modifications contribute to Hcy-regulated gene expression. Previous study demonstrates that TGF-β increases the recruitment of SET7/9, a H3K4 mono-methyltransferase, to the promoter of *Col1α1*, increases H3K4me1 level at the promoter of *Col1α1*, results in *Col1α1* gene induction in rat mesangial cells [26]. Moreover, histone deacetylase inhibitor trichostatin A abrogated the stimulatory effect of TGF-β on collagen I transcription in skin fibroblasts [27,28] and renal proximal tubular cells [29]. Since TGF-β1 is upregulated in the liver of HHcy mice, it is possible that TGF-β-induced histone modifications also contributes to HHcy-modulated ECM genes expression.

G9a, a key methyltransferase responsible for H3K9me2 at euchromatin and facultative heterochromatin, does not contain a DNA binding sequence [30]. Previous studies demonstrate that recruitment of G9a by NRSF is important in the repression of neuronal genes outside of the nervous system [31,32]. In the present study, we found, for the first time, that there is a NRSE in the promoter of *COL1A1*, and G9a is recruited to the *COL1A1* promoter via the NRSE. This finding adds *COL1A1* gene as a new member to the family of NRSE-containing genes, and supports the role of NRSF in controlling large programs of transcriptional regulation.

G9a is widely expressed in most tissues including fetal liver, bone marrow, peripheral blood leukocytes, thymus, lymph node, spleen and developing skeletal muscles [33]. Cancer transcriptome analysis revealed that G9a is overexpressed in many different types of tumors including hepatocellular, colon, prostate, lung and invasive transitional cell carcinomas and in B cell chronic lymphocytic leukemia, and responsible for various aspects of tumorigenesis, including cellular differentiation, proliferation and epithelial to mesenchymal transition [34]. Therefore, it is important to identify factors that could modulate the expression of G9a. Previous study showed that repeated administration of cocaine decreases G9a mRNA levels in nucleus accumbens, and results in increased gene expression [35,36]. In the present study, we identified Hcy as another factor which inhibits G9a gene expression. Previous study reported that Hcy accelerates protein degradation [37,38]. We treated LO2 cells with proteasome inhibitor MG132, and lysosome inhibitor chloroquine, but did not observed any obvious effect on Hcy-induced G9a suppression (data not shown), indicating that Hcy does not promote G9a degradation. We next measured mRNA level of G9a and found that Hcy decreased G9a mRNA in a time and dose-dependent manner, suggesting that Hcy modulates G9a expression at the transcription level.

The transcription regulation of G9a has been much less explored. Previous study showed that transcription factor C/EBPβ binds to the promoter of G9a, and C/EBPβ activates G9a expression during preadipocyte differentiation. Increased G9a expression results in enhanced H3K9 dimethylation at the C/EBPα promoter, which in turn represses C/EBPα expression [39]. Using the online prediction service of NCBI, we found 6 potential binding sites of C/EBPα in the proximal promoter region (from -1000bp to the transcription start site) of G9a gene. It is therefore possible that HHcy downregulates G9a expression through regulating the expression of C/EBPα and/or C/EBPβ. In supporting this hypothesis, previous studies have shown that both C/EBPα and C/EBPβ are expressed at high levels in the liver and play decisive roles in hepatocyte proliferation and differentiation [40]. Moreover, the *cebpa* promoter region contains CpG islands and *cebpa* gene expression is elevated in *dnmt1*-deficient embryos [41]. Since increased Hcy elevates the level of SAH which inhibits transferring methyl group to acceptors, Hcy might induce C/EBPα expression through inhibiting DNA hypomethylation at the promoter of C/EBPα, which in turn modulates G9a expression. Noteworthy, we found that Hcy treatment caused a more dramatic decrease in G9a protein than that of mRNA, suggesting that post-transcriptional mechanism is also involved in Hcy-modulated G9a expression.

Hcy-induced DNA hypomethylation has been proposed as a biochemical mechanism by which Hcy modulates gene expression. However, results have been inconsistent in many animal studies. Hcy increased cellular SAH concentration in endothelial cells, but not in vascular smooth muscle cells [42]. Moreover,in CBS null mice, higher SAH concentrations were detected in all tissues studied, but lower DNA methylation status was only detected in the liver [43]. These studies suggest other mechanisms might be implicated in Hcy-modulated gene expression. Our study showed that Hcy downregulated the expression of histone methyltransferase G9a, which in turn decreased the level of H3K9me2 at the promoter of *COL1A1*, suggesting the involvement of histone modifications in Hcy-modulated gene expression. In line with our finding, previous study demonstrated that Hcy reduced the binding of methyl CpG binding protein 2 (MeCP2) and increased the bindings of acetylated histone H3 and H4 at the cyclin A promoter [15]. Increasing evidence demonstrates that H3K9 hypermethylation serves as a docking site for the chromatin modifier protein heterochromatin protein 1(HP1), which in turn recruits DNMT1 and stimulates its activity leading to DNA hypermethylation in the surrounding area [44,45]. By using the online prediction service of NCBI, we found a couple of CpG islands in the promoters of both *COL1A1* and *Col1α1* gene. Therefore, Hcy-mediated G9a repression might lead to DNA hypomethylation to reinforce the epigenetic activation of gene transcription. Consistently, it has been shown that disrupting the function and expression of G9a by its inhibitor and siRNA resulted in a marked reduction in DNA methylation at the COX-2 promoter and led to the restoration of COX-2 expression [46,47]. Conversely, co-recruitment of G9a, DNMT1, and HP1 to the promoter of the survivin gene stimulates H3K9me2 and DNA hypermethylation [44,45].

Taken together, our study showed that Hcy downregulates G9a expression, which in turn decreases the binding of G9a and the level of H3K9me2 on the promoter collagen I gene, leads to its upregulation. Because G9a specifically catalyzes H3K9me2, it is conceivable that Hcy-mediated G9a repression causes a decrease in global euchromatic H3K9me2, which might alter the expression of numerous genes. Therefore, it will be of great interest to further explore the involvement of G9a-mediated histone modification in HHcy-induced multi-organs damage.

## Materials and Methods

### Cell culture and treatment

Human hepatic cell lines (LO2) (American Type Culture Collection, Manassas, VA) were cultured in DMEM supplemented with 10% FBS (Life Technologies, Gaithersburg, MD) at 37°C in a 5% CO_2_ incubator. Fresh medium containing DL-Hcy was replaced every 24 h. DL-Hcy was purchased from Sigma-Aldrich (St Louis, MO).

### Plasmid constructs

Human G9a were amplified and subcloned into pcDNA3.1 vector. To generate pCol-GL3 reporter plasmid, a fragment containing 1500bp upstream of the transcriptional start site of human *COL1A1* promoter was amplified and subcloned into pGL3 vector (Promega, Madison, WI). Primers used were as follow: Forward 5’-CCCGGTACCAGAGAAATGAACAGGGCA-3’, Reverse 5’-CCCCTCGAGACTGGCCCGGGCCCCTTT-3’. All expression constructs were generated by standard PCR-based cloning strategies, and all expression constructs were verified by DNA sequencing.

### RNA Interference

Oligonucleotide siRNA duplex was synthesized by Shanghai Gene Pharma (Shanghai, China). RNAi oligonucleotides were transfected into LO2 cells using the Lipofectamine 2000 (Invitrogen, Carlsbad, CA) according to the manufacturer’s instructions. The sequences of RNAi oligonucleotides were as follows: scramble siRNA: UUCUCCGAACGUGUCACGU; G9a siRNA: CCAUGCUGUCAACUACCAUGG

### Real-time PCR

Total RNA was extracted from cells or tissues using Trizol reagent (Invitrogen,Carlsbad, CA). cDNA was synthesized from 2μg of RNA using the SuperScript kit (Invitrogen). Reaction was performed on a 7500 Sequence Detection System (Applied Biosystems). Primers used in this study were as follows:

human G9a Forward 5’-ACAGAGGAAGAGGTAGGCCC-3’, Reverse 5’-CCATGAACTCTCTCGGTGGC-3’;
human *COL1A1*: Forward 5’-GAGATGATGGGGAAGCTGGA-3’, Reverse 5’-GCACCATCATTTCCACGAGC-3’;
human GADPH Forward 5’-AGAAGGCTGGGGCTCATTTG-3’, Reverse 5’-AGGGGCCATCCACAGTCTTC-3’;
mouse G9a Forward 5’-TTCCTTGTCTCCCCTCCCAG-3’, Reverse 5’-CTATGAACTCTCTCGGCGGC-3’;
mouse *Col1α1*: Forward 5’-GAGAGGTGAACAAGGTCCCG-3’, Reverse 5’-AAACCTCTCTCGCCTCTTGC-3’;
mouse GADPH Forward 5’-GGTGAAGGTCGGTGTGAACG-3’, Reverse 5’-CTCGCTCCTGGAAGATGGTG-3’.

### Western blotting

Cells and liver tissues were lysed with immunoprecipitation assay buffer (25mM Tris-HCl, pH 7.4, 150mM KCl, 5mM EDTA, 0.5% Na deoxycholate, 0.1% SDS, 1% NP-40). Lysates were subjected to Western blotting using method described previously [19]. The following primary antibodies were used in this study: anti-G9a (Abcam ab40542), anti-Col I (Abcam ab34710), anti-dimethyl-H3K9 (Abcam ab1220) and β-actin (Huatesheng Biotechnolgy, Fushun, China).

### ChIP assay and q-ChIP PCR

Chromatin immunoprecipitation (ChIP) assay was performed as described previously [19]. Briefly, 2×10^7^cells were fixed with 1% formaldehyde at 37°C for 10 min and were then lysed on ice for 15 min. These lysed extracts were subjected to shearing by sonication. After centrifugation at 14,000 rpm for 15 min, the soluble chromatin was subjected to immunoprecipitation with indicated antibodies, and then the complexes were drawn off with protein A-agarose beads and washed sequentially with low-salt, high-salt, LiCl, and Tris-EDTA buffers and were finally extracted with freshly prepared 1% SDS-0.1 M NaHCO_3_. Heating the samples at 65°C for 6 h, and then DNA was purified with a Qiagen DNA extraction kit. Primers used in this study were as follows: human *COL1A1*: Forward 5’-GCTGGGAAGGAGGGTCTCTA-3’, Reverse 5’-TGAGAGATGGAGTGGGGAGG-3’; mouse *Col1α1* Forward 5’-CATGGCCAGGAGGACCTTTT-3’, Reverse 5’-TTGATGGAGAGCTGGGAGGA-3’.

### Animals and Experimental Design

Wild-type C57BL/6 mice, obtained from and housed in Southern Medical University animal facility,at the age of 6–9 weeks, were fed with either standard rodent chow or HM diet containing 19.56g/kg (2%) methionine and sufficient basal levels of B vitamins [22]. After 2 weeks on the diet, mice were sacrificed. Blood was collected by cardiac puncture. Liver tissues were removed, flash-frozen, and stored at -80°C. All experiments were approved by the Southern Medical University Ethics Committee for Animal Experiments and strictly adhered to the guidelines for animal experiments of Southern Medical University. All surgery was performed under pentobarbital sodium, and all efforts were made to minimize suffering.

### Immunohistochemistry

Liver was fixed overnight in 4%paraformaldehyde solution in phosphate-buffered saline. Sections (4 μm thickness) were deparaffinized with xylene, followed by rehydration in ethanol. Hydrogen peroxide (3%) was used to eliminate endogenous peroxidase. Sections were incubated overnight at 4°C with primary antibodies against Col I (Abcam 34710). After extensive washing in PBS buffer, sections were then incubated for 30 minutes with secondary antibodies (Dako, Carpinteria, CA). The immunostaining was examined by an Olympus BX51 microscope (Olympus, Tokyo, Japan). Positive stains were quantified using image analysis software (Image Pro-Plus, Media Cybernetics, Silver Spring, MD).

### Masson Trichrome Staining

Selected liver sections were stained using the Masson Trichrome Stain Kit (Richard-Allan Scientific, Kalamazoo, MI) according to the manufacturer’s protocols.

### Hcy measurement

Total plasma Hcy concentration was measured using Homocysteine-EIA Kit (Axis Shield, Scotland) according to the manufacturer’s instructions. Hcy measurement was calibrated to the NIST standard reference material SRM1955.

### Luciferase reporter assays

LO2 cells were transfected with pCol-GL3 reporter plasmid, pRL null together with siG9a or Flag-tagged G9a using Lipofectamine 2000 (Invitrogen). Cells were harvested 48 h after transfection and the luciferase activities were analyzed by the luciferase reporter assay system (Promega, Madison, WI) on a GloMax 96 MicroplateLuminometer (Promega). Renilla luciferase activity was normalized to firefly luciferase expression for each sample.

### Statistical analyses

Data were expressed as means ± SD. Comparisons between two groups were conducted using the two-tailed *t* test. Differences among more than two groups were compared using one-way ANOVA. *P*<0.05 was considered statistically significant.
